# Association Between the Ambient Fine Particulate Pollution and the Daily Internal Medicine Outpatient Visits in Zhoushan, China: A Time-Series Study

**DOI:** 10.3389/fpubh.2021.749191

**Published:** 2021-10-26

**Authors:** Wen-Yi Liu, Jing-Ping Yi, Tao-Hsin Tung, Jian-Bo Yan

**Affiliations:** ^1^Department of Health Policy Management, Bloomberg School of Public Health, Johns Hopkins University, Baltimore, MD, United States; ^2^Shanghai Bluecross Medical Science Institute, Shanghai, China; ^3^Institute for Hospital Management, Tsing Hua University, Beijing, China; ^4^Zhoushan Municipal Center for Disease Control and Prevention, Zhoushan, China; ^5^Evidence-Based Medicine Center, Taizhou Hospital of Zhejiang Province Affiliated to Wenzhou Medical University, Linhai, China

**Keywords:** outpatient visit, PM_2.5_, SO_2_, China, Zhoushan, air pollution, time-series study

## Abstract

**Background:** There has been a recent worsening of air pollution in China, which poses a huge threat to public health by inducing and promoting circulatory and respiratory diseases. This study aimed to explore the association between the concentration of air pollution and daily internal medicine outpatient visits registered for the treatment of circulatory and respiratory symptoms in Zhoushan, China using a time-series method.

**Methods:** We validated and acquired the daily internal medicine outpatient visits records between January 1, 2014, and December 31, 2019, from the Zhoushan Center for Disease Control and Prevention in Zhejiang, China. Further, we collected the daily average records of the ambient air pollutants from the Zhoushan Environmental Monitoring Centre within the same duration. A generalized additive model with the natural splines was constructed to explore the association between the ambient air pollutants and daily internal medicine outpatient visits. Further, we conducted a lag analysis by using the distributed lag non-linear model to estimate the time-delayed effects of the air pollutants on the daily internal medicine outpatient visits.

**Results:** A total of 2,190,258 daily internal medicine outpatient visits with a mean of 202.4 visits per day were recorded. The non-linear relationships were found among particulate matter_2.5_ (PM_2.5_), sulfur dioxide (SO_2_), and the daily internal medicine outpatient visits. Overall, PM_2.5_ was positively correlated with the daily internal medicine outpatient visits. Both ozone (O_3_) and SO_2_ had significant delayed effects on the daily internal medical outpatient numbers; however, PM_2.5_ only showed a short-term risk.

**Conclusion:** Short-term exposure to PM_2.5_ was associated with an increase in the daily internal medicine outpatient visits for circulatory and respiratory diseases/symptoms in Zhoushan, China. SO_2_ and O_3_ were shown to induce significant effects after a concentration-dependent time lag.

## Introduction

China has recently embarked on large-scale urbanization and industrialization, which have posed serious environmental challenges (1). In comparison to the other environmental pollution conditions, the frequent air pollution incidents have received increasing attention since the severe smog pollution observed in 2013, which affected 75% of the Chinese cities and 800 million people (2). Air pollution is a major contributor to the global burden of disease, especially in relatively low-income countries; moreover, it is ranked fourth in terms of the age-standardized rates of the disability-adjusted life years (3). In China, circulatory and respiratory diseases represent substantial healthcare burdens. Specifically, cerebrovascular disease and ischemic heart disease are the most common causes of mortality (4). Respiratory infections adversely affect the quality of life; moreover, chronic respiratory diseases accounted for 9% of all the deaths from non-communicable diseases in China in 2016. Exposure to ambient air pollution can have serious adverse health effects and is considered a risk factor for acute or chronic respiratory diseases including chronic obstructive pulmonary disease (5, 6). Additionally, there have been numerous studies on the association of environmental pollution with morbidity and mortality due to circulatory diseases including heart disease (7, 8) and stroke (9).

Air pollution is a potential risk factor that in conjunction with the high outdoor activity levels that can further produce greater health threats and, thus, requires policy-based monitoring. Therefore, determining the relationship between air quality and disease burden is of public health importance. Previous Chinese studies have reported a significant relationship between air pollution and daily internal medicine outpatient visits for the single-system (circulatory or respiratory) diseases; moreover, they have demonstrated the disparities among the different air pollutant types and geographical locations (9–12). Most studies have been conducted in the northern and western regions of China, which have severe air pollution, with only few studies being conducted in the southern coastal areas (13–15). However, the association of air pollution with the combination of circulatory and respiratory adverse events remains unclear, especially for pollutants other than particulate matter (PM).

Zhoushan is a coastal city in southern China with a subtropical monsoon climate and relatively good air quality compared to the other cities in the area. At the end of 2018, the average PM_2.5_ concentration in Zhoushan was 22 μg/m3; further, 94.8% of the days had good air quality. This study aimed to use the official air pollution monitoring data published by the National Air Quality Monitoring Network and the internal medical outpatient records (including cardiovascular, cerebrovascular, and respiratory diseases/symptoms) in Zhoushan to evaluate the relationship between short-term exposure to air pollution and multisystem diseases from 2014 to 2019.

## Methods

### Data Collection

This study was conducted at the Zhoushan Hospital, which is the only tertiary medical center in Zhoushan City. Daily internal medicine outpatient visits records from January 1, 2014, to December 31, 2019, were acquired from the Zhoushan Center for Disease Control and Prevention in Zhejiang, China. The reason for seeking medical attention after diagnosis was recorded based on the 10th edition of the International Classification of Diseases. The study outcomes (internal medical diseases) included circulatory diseases (I00–I99), respiratory diseases (J00–J99), and circulatory and respiratory symptoms (R00–R09), which covered the vast majority of the circulatory and respiratory systems that triggered an outpatient visit.

We collected the daily average information regarding the ambient air pollutants from the Zhoushan Environmental Monitoring Centre over the same duration including the average daily levels of PM_2.5_, PM_10_, sulfur dioxide (SO_2_), nitrogen dioxide (NO_2_), carbon monoxide (CO), and ozone (O_3_). Further, we obtained the meteorological data within the same period from the China Meteorological Data Sharing Service System (http://zshbj.zhoushan.gov.cn/index.html).

### Ethical Considerations

This study was conducted following the principles of the Institutional Ethics Committee and in accordance with the Declaration of Helsinki. All the information of the participants was kept anonymous. This observational study was approved by the Institutional Review Board of Shanghai International Medical Center on April 15, 2021 (SIMC-IRB No: 20210513).

### Time-Series Model

A generalized additive model with the natural splines was used to estimate the relationship of the daily internal medicine outpatients with the ambient air pollutant concentrations (e.g., PM_2.5_, PM_10_, SO_2_, O_3_, NO_2_, CO) and meteorological factors (temperature, relative humidity, and atmospheric pressure). Time-related natural spline functions and meteorological variables were applied to the generalized additive model after adjustment for the confounding factors. The Spearman's test was used to examine between the variable correlations to prevent multicollinearity.

The Akaike's information criterion was examined for the model fitting. Moreover, we used the partial autocorrelation equations to assess the residual autocorrelations. To synthesize the findings from Akaike's information criterion, the partial autocorrelation function, three degrees of freedom (df) for the relative humidity, and six df for the mean temperature were included. The model is described as follows:


$$\begin{array}{l} log[E\left(Y_{t}\right)]=Intercept+ns\;\left(time,18\right)+ns\;\left(temperature,6\right)\\ \;+ns\;\left(humidity,3\right)+DOW+\sum_{i\;=\;1}^{q}\beta_{i}(X_{i}) \end{array}$$

where *E*(*Y*_*t*_) is the expected number of the daily internal medicine outpatient visits on day t, β_*i*_ is the log-relative rate of the daily internal medicine outpatient visits related to the air pollutant concentration, *X*_*i*_ represents the air pollutant concentration at day t, DOW is the dummy variable for the weekdays, and ns (time/temperature/humidity, 18/6/3) is the natural spline equation for the temperature and humidity with 18/6/3 df. We assessed the relative risks and CIs to determine the effects of the different air pollutants on the daily internal medicine outpatient visits. All the estimations were presented as relative risks and 95% CI of the daily internal medicine outpatient visits related to per unit increase in the concentration of the ambient air pollutants.

We used the distributed lag non-linear model (DLNM), including the quasi-Poisson regression, to identify the lag effect of the ambient air pollutants on the daily internal medicine outpatient visits. The DLNM model (16) was as follows:


$$\begin{array}{l} log[E\left(Y_{t}\right)]=Intercept+ns\;\left(time,18\right)+ns\;\left(temperature,6\right)\\ \;+ns\;\left(humidity,3\right)+DOW+cb\;(X_{i},lag=10) \end{array}$$

where: *cb* is the cross-basis function, which simultaneously specifies the exposure–lag–response relationship in the exposure–response and lag–response dimensions.

Subsequently, we conducted a lag analysis by using the single-day lag (0–7) to determine the delayed consequences of the air pollutants on the daily internal medicine outpatient visits.

### Statistical Analysis

The statistical analyses were performed by using the R software (version 4.0.4, Free Software Foundation's GNU General Public License) with the “mgcv” and “DLNM” packages. Statistical significance was set at *p* < 0.05. The results were presented as the percentage change of the daily internal medicine outpatient visits per unit increase of the air pollutants per day.

## Results

A total of 2,190,258 daily internal medicine outpatient visits (not including emergency department visits and hospital admissions) were recorded between January 1, 2014, and December 31, 2019. The daily average of the internal medicine outpatient visits was 202.4. The daily average temperature and humidity were 17.5°C and 80.9%, respectively. The annual number of the daily internal medicine outpatient visits was 369,482 (16.9%), 398,805 (18.2%), 361,345 (16.5%), 366,002 (16.7%), 392,752 (16.6%), and 301,872 (13.8%) for the 6 respective years (2014–2019). The mean air pollutant concentrations of CO, SO_2_, NO_2_, O_3_, PM_2.5_, and PM_10_ were 0.7 mg/m^3^, 7.1, 19.9, 94.0, 26.4, and 45.6 μg/m^3^ per day, respectively (Table 1).

**Table 1:** Descriptive statistics for the daily outpatient clinics, concentrations of air pollutants, and weather conditions.

	**2014**	**2015**	**2016**	**2017**	**2018**	**2019**	***P***
	**Mean (SD)**	**Mean (SD)**	**Mean (SD)**	**Mean (SD)**	**Mean (SD)**	**Mean (SD)**	
**Meteorological measure (24-h average)**							
Temperature (°C)	17.0 (7.6)	17.2 (7.4)	17.9 (8.1)	17.8 (8.1)	17.7 (8.1)	17.5 (7.4)	0.616
Humidity (%)	80.1(11.7)	80.8 (11.4)	82.3 (11.6)	79.0 (12.0)	81.9 (11.8)	81.3 (11.6)	0.002
**Main air pollutant concentrations (24-h average)**							
SO_2_ (μg/m^3^)	5.8 (4.6)	6.4 (3.7)	9.0 (2.9)	10.2 (2.9)	6.73 (3.3)	4.65 (1.5)	<0.001
NO_2_ (μg/m^3^)	22.2 (12.8)	23.4 (13.6)	19.9 (10.9)	18.0 (10.5)	17.55 (10.8)	18.3 (9.6)	<0.001
CO (mg/m^3^)	0.7 (0.2)	0.6 (0.3)	0.7 (0.2)	0.7 (0.2)	0.7 (0.2)	0.6 (0.2)	<0.001
O_3_ (μg/m^3^)	89.9 (32.1)	93.9 (32.4)	95.6 (35.2)	101.9 (34.6)	86.4 (32.8)	96.3 (31.3)	<0.001
PM_10_ (μg/m^3^)	57.0 (35.9)	49.4 (31.8)	44.4 (25.5)	46.0 (26.8)	39.6 (23.4)	37.1 (24.6)	<0.001
PM_2.5_ (μg/m^3^)	31.2 (20.7)	31.3 (23.0)	26.8 (17.6)	26.0 (16.8)	23.3 (16.6)	19.8 (14.5)	<0.001
Outpatients	1015.1 (351.5)	1092.6(393.4)	987.3 (401.8)	1002.8 (419.2)	1076.0 (443.8)	827.1 (585.6)	<0.001

Table 2 shows the correlation between the ambient air pollutants and meteorological parameters. PM_2.5_ was positively correlated with PM_10_ (*r* = 0.882). Temperature was negatively correlated with PM_2.5_ (*r* = −0.323), PM_10_ (*r* = −0.360), NO_2_ (*r* = −0.306), CO (*r* = −0.275), and SO_2_ (*r* = −0.104); however, it showed a significant positive correlation with ozone (*r* = 0.147). Further, the humidity was negatively correlated with all the air pollutants. The possible collinearity of the various independent variables is demonstrated by the correlations among the aforementioned indexes.

**Table 2:** The Spearman's rank correlation coefficient between the daily ambient air pollutant and meteorological parameters.

	**Temperature**	**Humidity**	**SO_2_**	**NO_2_**	**CO**	**O_3_**	**PM_10_**	**PM_2.5_**
Temperature	1.000							
Humidity	0.300^***^	1.000						
SO_2_	−0.104^***^	−0.325^***^	1.000					
NO_2_	−0.306^***^	−0.126^***^	0.242^***^	1.000				
CO	−0.275^***^	−0.079	0.358^***^	0.324^***^	1.000			
O_3_	0.147^***^	−0.277	0.147	0.001	0.065^***^	1.000		
PM_10_	−0.360^***^	−0.464^***^	0.343^***^	0.561^***^	0.460^***^	0.288^***^	1.000	
PM_2.5_	−0.323^***^	−0.295^***^	0.321^***^	0.594^***^	0.524^***^	0.278^***^	0.882^***^	1.000

*** *p < 0.01*.

**Figure 1:**
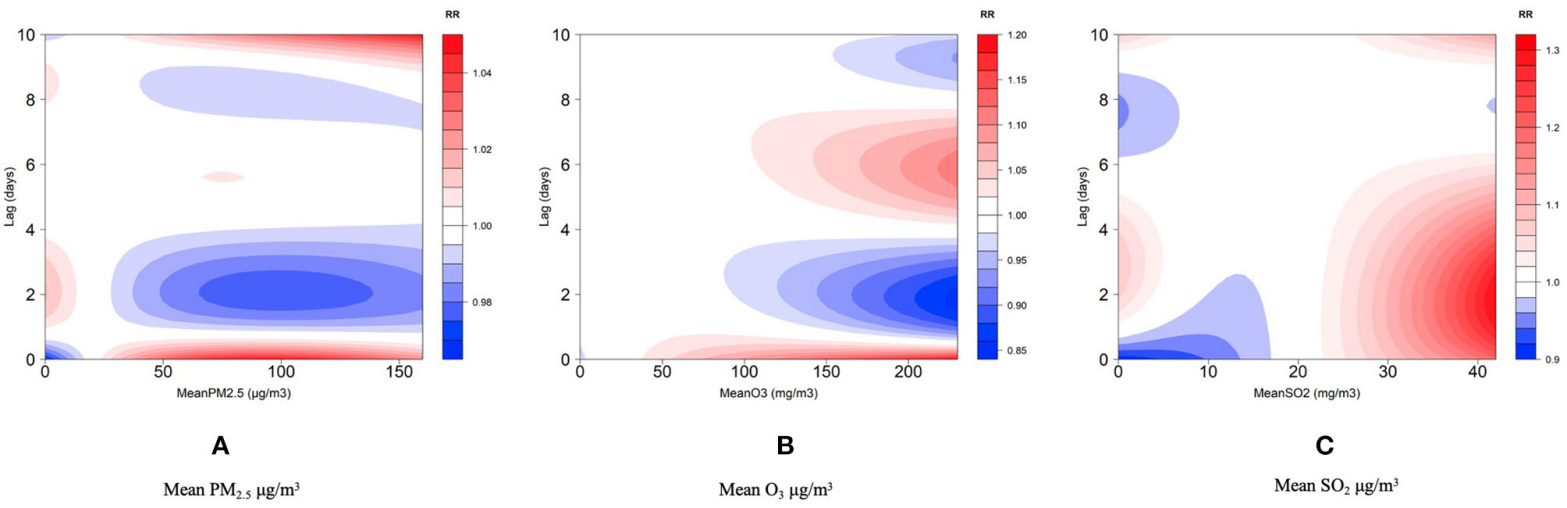
Single pollutant delay effect analysis results. **(A–C)**, respectively, show the analysis results of the single pollutant lag effect of particulate matter_2.5_ (PM_2.5_), ozone (O_3_), and sulfur dioxide (SO_2_). The horizontal axis is the concentration of the pollutants and the vertical axis is the number of days behind. A bar showing the effect on the daily internal medicine outpatient visits from blue (reduction) to red (increase).

Figure 1 demonstrates the lag effect of the ambient air pollutants and their non-linear relationship with the daily internal medicine outpatient visits. The estimated effects are presented as the mean and 95% CIs of the daily internal medicine outpatient visits per unit increase in PM_2.5_, SO_2_, or O_3_. All the highly concentrated air pollutants exhibited the hysteresis effects on the daily internal medicine outpatient visits with the significant among pollutant differences (*p* < 0.05). PM_2.5_, O_3_, and SO_2_ had significant delay effects on the daily internal medicine outpatient visits, with the delay effect of PM_2.5_, which is mainly a short-term risk, being relatively weak. SO_2_ and O_3_ had strong delay effects. The O_3_ concentration was positively correlated with the harmful effect in the very short term (1–2 days) and medium term (4–8 days). SO_2_ had a strong harmful effect within 6 days.

**Figure 2:**
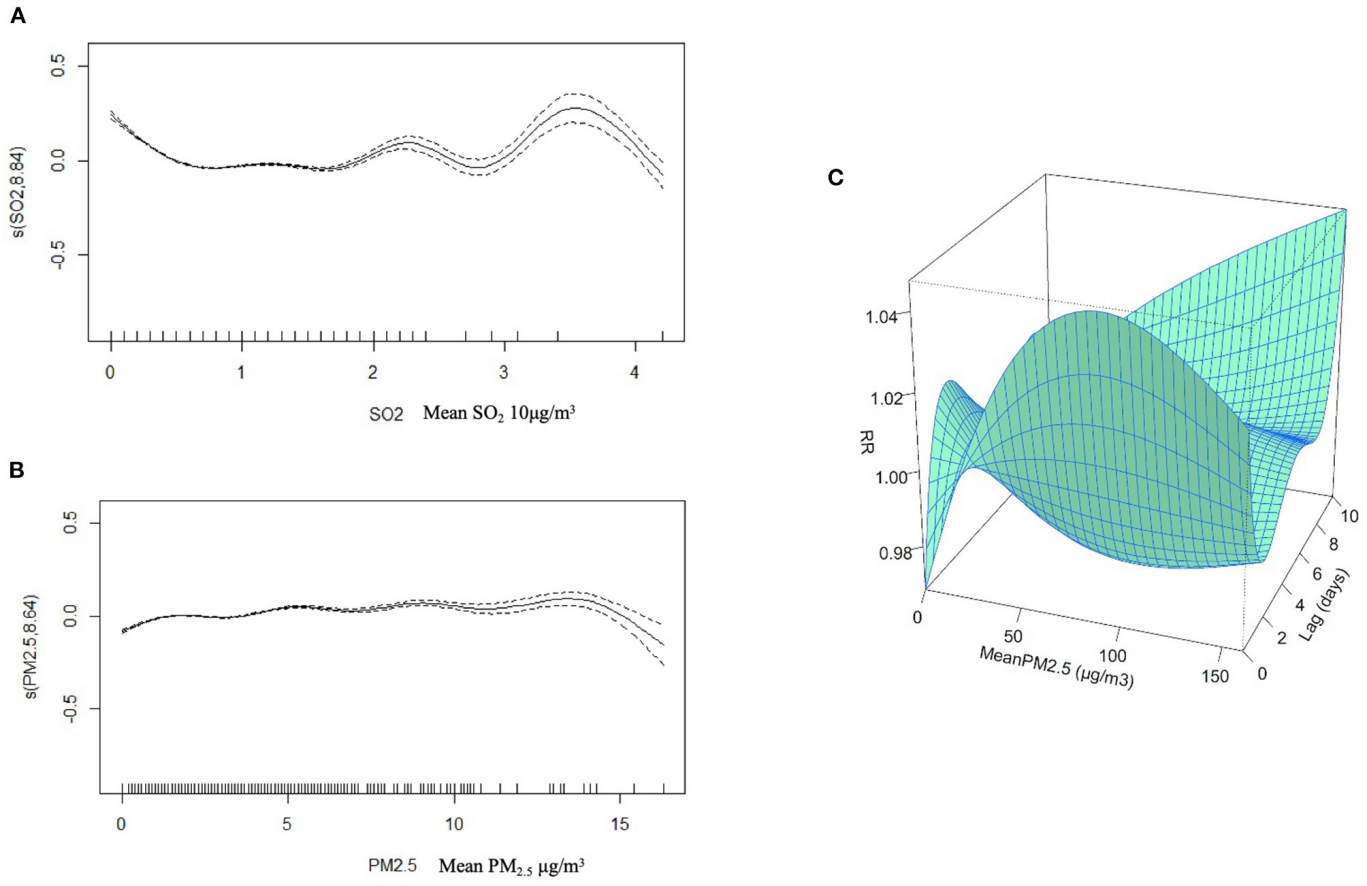
Non-linear relationship between the PM_2.5_ and daily internal medicine outpatient visits. **(A,B)** depict the visualization of the generalized additive model for SO_2_ and PM_2.5_. The solid curve in the middle represents a non-linear relationship between the independent variable (SO_2_ or PM_2.5_) and the outcome (outpatient visits). For the three-dimensional (3D) **(C)** plot, the X-axis represents PM_2.5_ concentration, the Y-axis represents the lag days, and the Z-axis represents the relative risk (RR).

Figure 2 shows the non-linear relationships among PM_2.5_, SO_2_, and the daily internal medicine outpatient visits. Overall, PM_2.5_ and SO_2_ were positively correlated with the daily internal medicine outpatient visits. Different relationship phases were observed. Specifically, when the concentration of SO_2_ is 22–28 and > 35 μg/m^3^, it shows that the influence is decreasing gradually; when the concentration of SO_2_ is between 28 and 35 μg/m^3^, it shows that the influence is increasing gradually. Generally, the curve of PM_2.5_ flattens out and the impact is decreasing gradually after the concentration of PM_2.5_ reaches 35 μg/m^3^. For the three-dimensional (3D) plot, we can see from the X-Z perspective that when the PM_2.5_ concentration is larger than 20 μg/m^3^, it is a risk factor and the risk levels fluctuate as the concentration grows. From the Y-Z perspective, we can identify a U-curve which tells on date lag 1 the negative effect is the greatest and on date lag 3 the negative effect reduces to the minimum. It indicates that the impact of PM_2.5_ is very short term. When the lag is 8–10, the effect again increases might be due to the increased concentration of PM_2.5_ on day 7. We cannot rule out the effect on day 7 because it is a mixed influence.

## Discussion

To the best of our knowledge, this is one of the very few Chinese studies to examine the relationship between air pollution and internal medical visits triggered by the symptoms related to the circulatory and respiratory systems of the patients. Moreover, this is the most recent short-term study to consider this issue. We chose Zhoushan City, a city with a stable climate and population, rather than a heavily polluted city in the north. This study further emphasizes that although air pollution is relatively light in the southern coastal areas, it still poses a public health threat and requires extra attention. We used the authoritative air pollution monitoring data published by the National Air Quality Monitoring Network after 2013 and set the observation cutoff date as 2019 to exclude the impact of the COVID-19 pandemic in and after 2020. The daily increase in the number of the daily internal medicine outpatient visits in Zhoushan Hospital was significantly related to the increase in PM_2.5_, O_3_, and SO_2_ levels. Moreover, the three pollutants showed different lag effects.

This study indicated that PM_2.5_ is a major risk factor for the daily internal medicine outpatient visits for circulatory and respiratory diseases and symptoms, which is consistent with the previous Chinese reports regarding single-system diseases (13–15). In Hong Kong, two studies reported a positive correlation of PM_2.5_ concentration with the hospital admissions for respiratory diseases (13, 14). In Beijing, a study finds that the short-term elevations in PM_2.5_ concentration may increase the risk of asthma exacerbations (17). Furthermore, national studies have reported that the short-term increase in PM_2.5_ concentrations is associated with the increased hospital admissions for cardiovascular diseases and pneumonia (18). An increase of 10 μg/m^3^ (lag 0–2) in the 3-day moving mean concentration of PM_2.5_ has been associated with a 0.31% (95% CI 0.28–0.46%) increase in the hospital admission for pneumonia (10). Furthermore, 10 μg/m^3^ increase in PM_2.5_ concentration has been associated with a 0.26% (95% CI 0.26–0.35%) and 0.34% (95% CI 0.34–0.48%) increase in the number of hospitalizations for cardiovascular disease (12) and ischemic stroke on the same day (9), respectively.

Several mechanisms underlie the relationship of PM with adverse circulatory and respiratory reactions. PM is a potent oxidant that produces free radicals, which may induce respiratory defense responses, promote oxidative stress in the lung cells, increase mucus secretion, and enhance bronchial reactivity (19, 20). Short-term exposure to high PM concentrations increases the level of reactive oxygen species in the lungs (21). This induces oxidative stress, which may impair the cellular defense and suppress the immune responses, and, therefore, increase susceptibility to the various infections (22, 23). Furthermore, PM concentration may adversely affect the vascular endothelial function, sympathetic nervous system activity, and systemic inflammation, which cause vasoconstriction as well as increased blood viscosity and the risk of thrombosis (24, 25). These are closely associated with various heart diseases and stroke.

Previous studies in South China have reported a positive correlation of SO_2_ and O_3_ with the number of daily internal medicine outpatient visits for respiratory and circulatory diseases. A study on the short-term effects of the air pollutants on the hospital admissions for the cardiovascular and respiratory diseases in Hong Kong between 1994 and 1995 reported that the relative risk of NO_2_ (μg/m^3^), O_3_ (μg/m^3^), SO_2_ (μg/m^3^), and PM_10_ (μg/m^3^) for the respiratory and cardiovascular hospital admissions ranged from 1.013 (SO_2_) to 1.022 (O_3_) and 1.006 (PM_10_) to 1.016 (SO_2_), respectively (26). Another study conducted in Ningbo, Zhejiang Province, reported a positive correlation between the average daily concentration of SO_2_ and the number of patients with upper respiratory diseases with an excess risk of 10–18% and 4–6 days lag effect for the different concentrations (27). A study in Hefei, Anhui Province, reported that for every 10 μg/m^3^ increase in the SO_2_ levels, the cardiovascular mortality rate increased by 5.26% at lag 3 (28). Additionally, studies conducted outside of China have also pointed to a positive relation between these air pollutants and several diseases. A study in South Korea reported that high levels of PM_10_, O_3_, and SO_2_ were significantly associated with an increased risk of atopic dermatitis, which triggers the daily internal medicine outpatient visits (29). Previous Western studies have reported an increased risk of cardiovascular and respiratory hospitalizations caused by the SO_2_ levels (30).

These contaminants increase the permeability of the epithelial cells by causing respiratory tract inflammation, overcoming the mucosal barrier, and triggering an allergen-induced response (31). SO_2_ and O_3_ aggravate the airway allergic responses to the inhaled allergens (32). O_3_ can induce neutrophil inflammation and increase the levels of several inflammatory mediators including prostaglandin E2. SO_2_ may induce bronchial constriction in patients with asthma; moreover, it may impair nasal respiration since the water-soluble gas SO_2_ is absorbed by the nasal mucosa (33). Additionally, several studies have demonstrated that asthma-related genes regulate airway inflammation and promote mucus hypersecretion after SO_2_ concentration (34, 35). Although the respiratory tract is the primary target of SO_2_ gas given its toxic effects, other organs and systems are also affected upon entry of this gas into the systemic circulation through the bloodstream (36, 37). Given the high water solubility of SO_2_, it is hydrated to form the sulfite and bisulfite anions, which are subsequently oxidized and have toxic effects on the other systems (31).

This study has several limitations. First, we only selected one city in China, which is not nationally representative; however, our results could significantly contribute to the local prevention and control policies. Second, due to the limited data access, we did not include the data from all the hospitals in Zhoushan; nonetheless, Zhoushan Hospital is a general hospital with the highest grade in the city. Third, we are not able to conduct the subgroup analysis to investigate the age, sex, and season-specific associations between the air pollutants and daily internal medicine outpatient visits due to the limitation of the data. Each subgroup population has a different susceptibility to air pollution. The dataset also limits us to examine the effects of air pollution on circulatory and respiratory diseases, respectively. The future study should progress the diagnosis and validate the record of the outpatient services to avoid misclassification. Fourth, we used the average air pollutant concentration at the fixed monitoring points to infer the exposure of an individual, which may cause the potential exposure misclassification given the lack of exposure information of an individual. Moreover, we did not account for indoor air pollution including cooking and secondhand smoke. Fifth, the information and biochemical conditions were not simultaneously collected; further, several unknown potential factors could have introduced bias in this population-based study. This study impeded analysis of the relationship between the biochemical levels and outpatient visits. Last but not the least, after the correlation analysis, there is a strong correlation among the meteorological factors. In order to avoid the multiple linear regression, we did not include the atmospheric pressure in the statistical analysis. In conclusion, this study indicates that short-term exposure to air pollution was associated with the increased daily internal medicine outpatient visits for circulatory and respiratory diseases and symptoms in the southern Chinese city of Zhoushan. Moreover, they demonstrate that PM_2.5_ is a short-term risk, while SO_2_ and O_3_ have significant lag effects.

## Data Availability Statement

The original contributions presented in the study are included in the article/supplementary material, further inquiries can be directed to the corresponding author/s.

## Ethics Statement

The studies involving human participants were reviewed and approved by the Institutional Review Board of Shanghai International Medical Center on April 15, 2021 (SIMC-IRB No: 20210513). Written informed consent for participation was not required for this study in accordance with the national legislation and the institutional requirements.

## Author Contributions

W-YL, J-PY, T-HT, and J-BY conducted the study and drafted the manuscript. W-YL and J-PY participated in the design of the study and performed the data synthesis. T-HT and J-BY conceived the study and participated in its design and coordination. All authors have read and approved the final manuscript.

## Conflict of Interest

The authors declare that the research was conducted in the absence of any commercial or financial relationships that could be construed as a potential conflict of interest.

## Publisher's Note

All claims expressed in this article are solely those of the authors and do not necessarily represent those of their affiliated organizations, or those of the publisher, the editors and the reviewers. Any product that may be evaluated in this article, or claim that may be made by its manufacturer, is not guaranteed or endorsed by the publisher.
